# Chemical Constituents with Leishmanicidal Activity from a Pink-Yellow Cultivar of *Lantana camara* var. *aculeata* (L.) Collected in Central Mexico

**DOI:** 10.3390/ijms20040872

**Published:** 2019-02-18

**Authors:** Ronna Delgado-Altamirano, Rosa Isela López-Palma, Lianet Monzote, José Delgado-Domínguez, Ingeborg Becker, José Fausto Rivero-Cruz, Nuria Esturau-Escofet, Pedro A. Vázquez-Landaverde, Alejandra Rojas-Molina

**Affiliations:** 1 Posgrado en Ciencias Químico Biológicas, Facultad de Química, Universidad Autónoma de Querétaro, Cerro de las Campanas s/n, Querétaro 76010, México; ti2dronna@hotmail.com; 2 Laboratorio de Investigación Química y Farmacológica de Productos Naturales, Facultad de Química, Universidad Autónoma de Querétaro, Cerro de las Campanas s/n, Querétaro 76010, México; 3 Laboratorio de Inmunoparasitología, Unidad de Investigación en Medicina Experimental, Facultad de Medicina, Universidad Nacional Autónoma de México, Hospital General de México “Dr. Eduardo Liceaga”, Dr. Balmis 148, Doctores, Ciudad de México 06720, México; rosaislp@gmail.com (R.I.L.-P.); ferd@unam.mx (J.D.-D.); becker@unam.mx (I.B.); 4 Departamento de Parasitología, Instituto de Medicina Tropical “Pedro Kourí”, Apdo Postal 601, Havana 10400, Cuba; monzote@ipk.sld.cu; 5 Departamento de Farmacia, Facultad de Química, Universidad Nacional Autónoma de México, Ciudad de México 04510, México; joserc@unam.mx; 6 Instituto de Química, Universidad Nacional Autónoma de México, Ciudad de México 04510, México; nesturau@iquimica.unam.mx; 7 Centro de Investigación en Ciencia Aplicada y Tecnología Avanzada del Instituto Politécnico Nacional, Unidad Querétaro, Cerro Blanco 141 Colonia del Cimatario, Querétaro 76090, México; pavazquez@ipn.mx

**Keywords:** *Lantana camara*, leishmanicidal activity, *Leishmania mexicana*, *Leishmania amazonensis*, triterpenes, lantanilic acid, camaric acid, lantadene B, bio-directed fractionation

## Abstract

*Lantana camara* (L.) is employed by several ethnical groups to treat numerous diseases. Although there are no ethnomedical reports on its use against leishmaniasis, organic extracts prepared from *L. camara* were shown to display leishmanicidal activity. In the present study, we carried out a bioassay-guided fractionation of the dichloromethane extract from Mexican *L. camara* in order to identify the compounds responsible for the leishmanicidal activity. Eighteen chromatographic fractions (FI–FXVIII) were evaluated in vitro against *Leishmania mexicana* and *L. amazonensis*. FII, FX, FXI, FXV, and FXVI showed significant activity against both *Leishmania* strains, the most potent of which was FXV. Eicosane (**1**), squalene (**2**), β-ionone (**3**), caryophyllene oxide (**4**), β-caryophyllene (**5**), hexanoic acid (**6**), tiglic acid (**7**), a mixture of lantanilic (**8**) and camaric (**9**) acids, and lantadene B (**10**) were identified and obtained from the active fractions and evaluated for their leishmanicidal activity. The mixture of lantanilic (**8**) and camaric (**9**) acids (79%/21%) was the most potent one (half maximal inhibitory concentration (IC_50_) = 12.02 ± 0.36 μM). This study indicates that this cultivar of *L. camara* has high potential for the development of phytomedicines or as a source of natural products, which might represent lead compounds for the design of new drugs against leishmaniasis.

## 1. Introduction

Leishmaniasis describes a group of tropical diseases caused by protozoans of the genus *Leishmania*, which are transmitted by the bite of sandflies from the genera *Phlebotomus* and *Lutzomyia* [1]. The clinical manifestations of these diseases are diverse and depend on several factors such as the *Leishmania* species [2], the immunological [3,4,5] and nutritional [6,7,8,9] status of the patient, and the biochemical composition of sandfly saliva [10]. These clinical characteristics can be summarized in three main groups: (1) cutaneous leishmaniasis (CL) which, despite being the most common, is limited to the bite site and can heal spontaneously; (2) mucocutaneous leishmaniasis (MCL), where the parasite migrates to mucosal tissue, mainly the nose, and destroys it [11]; (3) visceral leishmaniasis (VL), in which the parasite is lodged in the liver and spleen, where it causes an abnormal growth and organic failure and may be fatal if it is not treated opportunely [12]. This group of diseases spread to approximately 98 developing countries and it is estimated that about two million people are infected. However, its incidence is increasing due to various factors such as civil wars, migration, traveling to endemic areas, urbanization, deforestation and agriculture close to phlebotomine breeding sites, and climate change [13,14,15,16,17,18]. In Mexico, these diseases are distributed in 13 of the 32 states of the country, where cutaneous leishmaniasis caused by *L. mexicana* is the most common form.

Since the early 20th century, pentavalent antimonials, such as Glucantime® and Pentostam®, were used as the first-line drugs for treating leishmaniasis. Nevertheless, in regions where leishmaniasis is a severe health problem, *Leishmania* created resistance to these kinds of drugs [19]. Moreover, it was widely demonstrated that antimonial compounds produce cardiac arrhythmias, which can be fatal [20]. Amphotericin B, pentamidine, paromomycin, and miltefosine are used as second-line drugs, which include other disadvantages such as storage and formulation difficulties, high price, low availability, and teratogenicity [21,22]. Thus, the search of molecules that can be used as lead compounds to develop novel treatments against leishmaniasis continues. One of the strategies focuses on the study of medicinal plants employed in ethnomedical systems of different cultures. Those studies provided a wide variety of secondary metabolites with in vitro anti-leishmanial activity [22]. Some in vivo studies demonstrated the potential benefits of natural products for the treatment of cutaneous and visceral leishmaniasis. It was demonstrated that α-bisabolol, a monoterpene alcohol that is the major component of *Matricaria chamomilla* essential oil, is capable of successfully reducing the parasite load in preclinical studies of two leishmaniasis models (*L. tropica* and *L. infantum*) [23,24]. On the other hand, oleuropein decreased the parasite load in a model of visceral leishmaniasis caused by *L. donovani*. The mechanism of action via which this secoiridoid, isolated from *Olea europea*, exerts its leishmanicidal activity involves a rise in reactive oxygen species, a downregulation of the expression of *L. donovani* glutamate–cysteine ligase (LdGCLC), an upregulation of the mGCLC of mammals, and the polarization of the Th1 immune response, which augments the gene expression levels of IL-12β, IL-10, TGF-β1, IFN-γ, Tbx21, and GATA3 [25,26]. These results indicate the high potential of natural products and their immunological regulating ability to fight leishmaniasis.

In an effort to identify plant species that could represent potential sources of leishmanicidal compounds, our group carried out an exhaustive research of several bibliographic sources that report the ethnomedical use of medicinal plants by different ethnical groups, particularly those who live in the states where the greatest number of leishmaniasis cases occurred. Although Mexico is recognized for its floristic richness and its important tradition in herbal medicine, the documented ethnomedical reports on medicinal plants used for treating leishmaniasis are very scarce. Therefore, we assessed the in vitro leishmanicidal activity of extracts obtained from 10 medicinal plants used for the treatment of other parasitic diseases. The results derived from that evaluation showed that the dichloromethane extract prepared from the aerial parts of a pink-yellow cultivar of *L. camara* var. *aculeata* (L.) collected in central Mexico displayed significant activity against *L. amazonensis* promastigotes and amastigotes [27].

*Lantana camara* var. *aculeata* (L.) is a native plant from the American tropics that possesses a high capability for environmental adaptation, which allowed its domestication and cultivation in several parts of the world [28,29]. Moreover, in some countries such as India, South Africa, and Australia, this species was considered as an invasive noxious weed that poisons livestock of free grazing [30,31]. In addition to its use as an ornamental plant due to the beauty of its flowers and its easy handling, *L. camara* was employed by several ethnical groups throughout the world to treat a great variety of diseases and pathological conditions [27,32,33,34,35,36,37,38,39,40]. Although, there are no ethnomedical reports on the use of *L. camara* to heal leishmaniasis, some research groups demonstrated that organic extracts prepared from *L. camara* collected in different geographical locations display leishmanicidal activity against some *Leishmania* strains [27,41,42]. Previously, Begum et al. (2014) reported that a methanolic extract obtained from the aerial parts of a Pakistani specimen of a red cultivar of *L. camara* exhibited activity against *L. major* promastigotes [41]. It is well known that *L. camara* (L.) is a worldwide shrub that displays a high phenotypic variability, manifested mainly in the color of its inflorescences, which can be white, yellow, orange, red, pink, violet, or mauve [28,43,44,45]. It was reported that different cultivars of *L. camara* show variations in their phytochemical profiles, which are associated with the color of their flowers [46] and their geographical distribution [47]. Since the biological activity strongly depends on the chemical composition, it is expected that modifications in the chemical profile alter the biological activity induced by extracts obtained from different *L. camara* specimens. In this context, the aim of the present study was to characterize the main components responsible for the leishmanicidal activity elicited by the dichloromethane extract of a pink-yellow cultivar of *L. camara* var. *aculeata* (L.) collected in central Mexico.

## 2. Results and Discussion

### 2.1. Leishmanicidal and Cytotoxic Activity of the Crude Extract and the Chromatographic Fractions Obtained from *L. camara*

In the present study, we evaluated the leishmanicidal activity of the dichloromethane extract of *L. camara* var. *aculeata* (L.) collected in central Mexico on *L. mexicana* in order to determine if this *Leishmania* strain was susceptible to the effect of this extract, as previously shown for *L. amazonensis*, since both species belong to the same *Leishmania* complex (*Leishmania mexicana*). We found that *L. mexicana* showed greater resistance than *L. amazonensis* to the leishmanicidal effect of the dichloromethane extract from *L. camara*. In fact, the half maximal inhibitory concentration (IC_50_) elicited by the extract on this species was approximately twofold higher than that found for *L. amazonensis* (Table 1). It is a well-known fact that *Leishmania* species display great variability in their susceptibility to reference drugs [48,49,50,51]. Moreover, different strains within a *Leishmania* species present variations in their profile sensitivity to anti-leishmanial drugs [52]. To date, the mechanisms underlying this phenomenon remain to be elucidated. It is worth mentioning that, in the present study, the *L. camara* extract was more selective and approximately 100-fold more potent than Glucantime® on *L. mexicana*. Contrastingly, the leishmanicidal activity of a methanol extract prepared from a Pakistani specimen of *L. camara*, which exhibited an IC_50_ = 42.0 ± 0.1 μM against *L. major* promastigotes, similar to the one we found against *L. mexicana*, turned out to be significantly less potent than the reference drugs (pentamidine and amphotericin B) [41]. These findings evidence the potential of the extract obtained from the pink-yellow cultivar of *L. camara* for the development of an anti-leishmanial phytomedicine or as source of natural compounds with leishmanicidal activity.

The dichloromethane extract from *L. camara* was fractionated, using open-column chromatography, into 18 final fractions (FI–FXVIII) according to their chromatographic similarity. All the chromatographic fractions were evaluated on promastigotes of *L. mexicana* and amastigotes of both *Leishmania* species. Additionally, the cytotoxicity elicited by the fractions was assessed on peritoneal macrophages from BALB/c mice. The results from these evaluations are presented in Table 1.

FII, FX, FXI, FXV, and FXVI were the most active and selective against *L. mexicana* and *L. amazonensis*. It was proposed that, in screenings against infectious organisms, a crude extract or a fraction that exhibits IC_50_ values under 100 μg/mL and selectivity index (SI) values greater than 5 are very promising to be explored [27,53,54].

According to what was found for the crude extract, in general, *L. mexicana* was more resistant than *L. amazonensis* to the effect of the fractions. Nevertheless, FX, FXI, and FXVI were equally active against both *Leishmania* strains. Interestingly, the anti-leishmanial activity of these fractions was significantly higher on *L. mexicana* amastigotes as compared to promastigotes of this species (IC_50_ < 12.5 μg/mL). A similar trend was observed in the case of the crude extract, where FXV showed the most potent effect and the best SI on *L. mexicana* promastigotes.

A chromatographic analysis of the most active fractions (FII, FX, FXI, FXV, and FXVI) was carried out in order to find the compounds responsible for the biological activity. It is important to mention that FXVI showed a comparable chromatographic profile to that of FXV; however, due to its very low yield, FXV was selected for further analysis.

### 2.2. GC–MS and NMR Analysis for the Identification of the Active Compounds Contained in FII, FX, FXI, and FXV

The GC–MS analysis of FII allowed the identification of 55 compounds, which were classified as monoterpenes (4.18%), sesquiterpenes (22.69%), diterpenes (3.53%), triterpenes (19.57%), hydrocarbons (46.46%), and others (3.67%). The 10 major components present in FII were eicosane (**1**) (24.08%), squalene (**2**) (19.57%), octacosane (6.63%), β-ionone (**3**) (4.50%), α-curcumene (4.37%), dihydroactinolide (2.86%), caryophyllene oxide (**4**) (2.18%), tiglic acid (**7**) (2.18%), β-caryophyllene (**5**) (1.02%), and hexanoic acid (**6**) (1.00%). Commercially available compounds were purchased for their individual leishmanicidal evaluation.

On the other hand, chlorophylls were removed from fractions FX, FXI, and FXV. Subsequently, they were analyzed using HPLC-PDA. FX presented four chromatographic peaks (t_R_ = 8.5, 9.0, 15.75, and 17.4 min); FXI presented one peak (t_R_ = 15.75 min), and FXV showed two peaks (t_R_ = 8.5 and 9.0 min).

Repeated washes of FXI enabled obtaining 28 mg of a compound isolated as beige needles, which was analyzed using NMR. ^1^H, ^13^C, COSY, HSQC, and HMBC spectra interpretation led to the identification of this compound as lantadene B (**10**). NMR spectra (Figures S1–S5) and chemical shifts of Lantadene B (**10**) are summarized in the Supplementary Material.

The two chromatographic peaks from FXV were separated by repeated injections in HPLC-PDA, which afforded two white amorphous powders. NMR spectra interpretation revealed that these two compounds were two isomeric triterpenes: lantanilic acid (**8**) (t_R_ = 8.5 min, 18.5 mg) and camaric acid (**9**) (t_R_ = 9.0 min, 8.5 mg). However, since their yield was not enough to separately evaluate their leishmanicidal activity, the original mixture of these triterpenes was used for this purpose. NMR analysis of this mixture evidenced that it consisted of 79% lantanilic acid (**8**) and 21% camaric acid (**9**). NMR spectra of both compounds are reported in Delgado-Altamirano et al. (2019) [55], their chemical shifts are summarized in the Supplementary Material.

**Figure 1:**
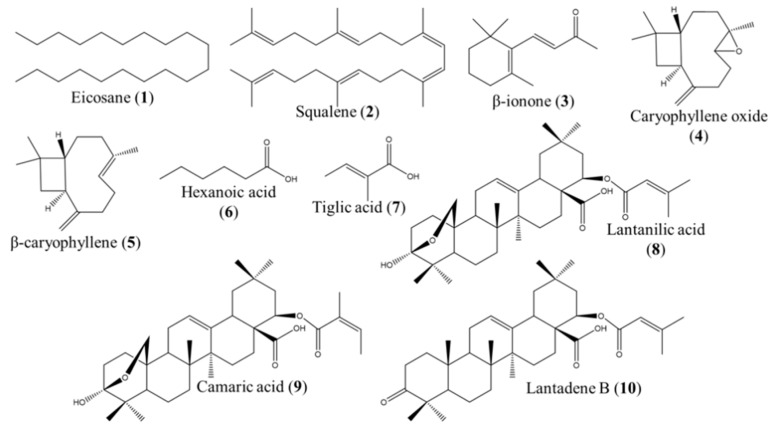
Compounds identified in the most potent fractions obtained from the dichloromethane extract of *Lantana camara*.

Three of the four chromatographic peaks from FX corresponded to lantanilic (**8**) and camaric (**9**) acids and lantadene B (**10**). Nevertheless, the compound eluted at t_R_ = 17.4 min could not be identified. The chemical structures of the identified compounds from fractions FII, FX, FXI, and FXV are presented in Figure 1.

### 2.3. Leishmanicidal Activity of the Compounds Identified in the Fractions FII, FX, FXI, and FXV

The major components contained in the active fractions were tested for their leishmanicidal activity on *L. mexicana* promastigotes. The IC_50_ values corresponding to the evaluated compounds are presented in Table 2.

Three of the tested compounds, caryophyllene oxide (**4**), β-caryophyllene (**5**), and lantanilic acid (**8**), were previously evaluated on other *Leishmania* species, whereas the present study represents the first report of the anti-leishmanial activity of eicosane (**1**), squalene (**2**), β-ionone (**3**), hexanoic acid (**6**), tiglic acid (**7**), camaric acid (**9**), and lantadene B (**10**).

Caryophyllene oxide (**4**) was extensively studied by Monzote and collaborators, who evaluated it against *L. amazonensis* (MHOM/BR/77/LTB0016; IC_50_ = 22.2 ± 10.4 μM for promastigotes; IC_50_ = 20 ± 1.8 μM for amastigotes) [56] and *L. tarentolae*, a *Sauroleishmania* species, (P10; IC_50_ = 36.0 ± 17.6 μM for promastigotes) [57]. They proposed that the leishmanicidal mechanism of action of this compound involved an enhancement of superoxide radicals, a decrease in promastigote oxygen consumption, and the inhibition of the mitochondrial electron transport chain complex III [57].

Taking into account the IC_50_ values obtained for the leishmanicidal activity of caryophyllene oxide (**4**) on *L. amazonensis*, *L. tarentolae*, and *L. mexicana*, this last species was shown to be the most resistant to this sesquiterpene (IC_50_ = 81.62 ± 2.16 μM, see Table 2), which confirms that, despite the fact that *L. amazonensis* and *L. mexicana* belong to the same complex, they have different drug susceptibilities.

A pure compound is considered active as an anti-infective when its IC_50_ value is lower than 25 μM [53]; therefore, caryophyllene oxide (**4**) was considered active against *L. amazonensis* and *L. tarentolae*, but not against *L. mexicana*. In addition, Monzote and collaborators tested caryophyllene oxide (**4**) on an in vivo model of cutaneous leishmaniasis caused by *L. amazonensis* in BALB/c mice. They administered an intralesional dose of 30 mg/kg/day for 14 days and observed a 74% reduction in the lesion size, as compared to the control, while Glucantime® only decreased the lesion size by 60%. Caryophyllene oxide (**4**) also diminished the parasite load by 90%, while Glucantime® only decreased it by 50% [58]. These data indicated the high value of caryophyllene oxide (**4**) as a leishmanicidal agent for the development of drugs to treat cutaneous leishmaniasis. It is necessary to evaluate other routes of administration in addition to the intralesional one in order to demonstrate whether this compound has the ability to heal leishmaniasis when administered orally or topically, which would represent an enormous advantage when developing a drug, since the lack of adhesion to treatment, due to the difficulty of administration, is one of the main problems of leishmaniasis therapy [59].

β-caryophyllene (**5**) was another sesquiterpene detected in the dichloromethane extract of *L. camara*. Soares and collaborators found that this compound was the main constituent of the *Copaiba* spp. oil, which exhibited significant leishmanicidal activity against *L. amazonensis* (WHOM/BR/75/Josefa) (IC_50_ = 6.36 μM; SI = 49) [60]. A comparison of this result with ours (IC_50_ = 74.4 ± 4.4 μM) confirmed once again that *L. mexicana* is more resistant than this *L. amazonensis* strain. At present, the leishmanicidal mechanism of action of β-caryophyllene (**5**) remains to be elucidated, as well as its effect on in vivo models.

**Figure 2:**
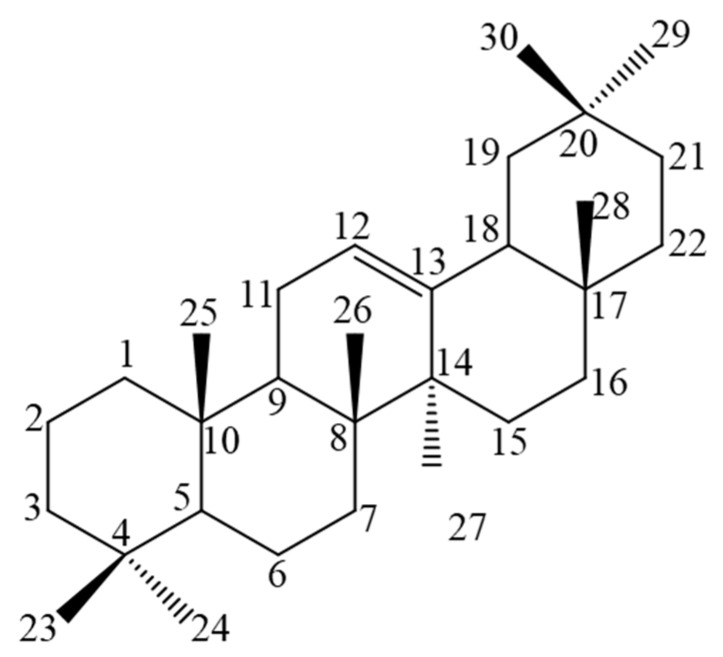
Oleanane core of active triterpenes from *L. camara*.

Of all the compounds detected in the bioactive fractions obtained from the dichloromethane extract of the pink cultivar of *L. camara*, the pentacyclic oleanene-type triterpenes (oleanane-core, Figure 2), lantadene B (**10**), and the mixture of lantanilic (**8**) and camaric (**9**) acids were the most active. Contrastingly, Begum and collaborators isolated four oleanane- (lantaninilic acid, oleanolic acid, lantadene A, and lantanilic acid (**8**)), three ursane- (lantoic acid, ursolic acid, and camarinic acid) and one lupane-type (betulinic acid) triterpenes from the methanolic extract of a cultivar of *L. camara* collected in Pakistan. They evaluated the in vitro leishmanicidal activity of those eight triterpenes against *L. major* promastigotes, and found that ursolic acid (IC_50_ = 12.4 ± 0.03 μM) was the most potent. Regarding the oleanane triterpenes obtained from the Pakistani specimen of *L. camara*, lantanilic acid (**8**) (IC_50_ = 21.3 ± 0.02 μM) and lantadene A (IC_50_ = 20.4 ± 0.01 μM) were shown to be the most potent, whereas oleanolic acid (IC_50_ = 53.0 ± 0.02 μM) and lantaninilic acid (IC_50_ = 164.0 ± 0.8 μM) were significantly less potent [41]. In the present study, no statistically significant differences were found between the IC_50_ values for the lantanilic (**8**) and camaric (**9**) acid mixture and lantadene B (**10**). However, the IC_50_ of the mixture on *L. mexicana* promastigotes was approximately twofold more potent than that of lantadene B (**10**). Lantanilic acid (**8**) and lantadene B (**10**) share a 3,3-dimethylacrylate moiety at C-22, whereas camaric acid (**9**), an isomer of lantanilic acid (**8**), bears a *trans*-2,3-dimethylacrylate. The main structural difference between the triterpene acids and lantadene B (**10**) is the presence of a hydroxyl and an ether group at position C-3. Considering this structural feature, it can be hypothesized that the presence of this hydrogen-bond donor in lantanilic acid represents a pharmacophore element. This hypothesis is supported by the findings of previous studies which involved the evaluation of the anti-leishmanial activity of other natural oleanane triterpenes isolated from different plant species.

Ridoux and collaborators analyzed the activity of three oleanane saponins isolated from *Hedera helix* (L.), α-hederine, β-hederine, and hederacolchiside A_1_, against *L. mexicana* (MHOM/MX/95/NAN1). Hederacolchiside A_1_ elicited the most potent leishmanicidal activity, followed by α-hederine and β-hederine [61]. Within this group of compounds, the number of sugar units bonded to C-3 significantly influences the anti-leishmanial effect, since hederacolchiside A_1_, which possesses a trisaccharide group, shows greater activity than the other two saponins that only bear a disaccharide portion. The only difference between α- and β-hederine is the presence of a hydroxyl group at C-24, which is spatially close to C-3. These findings suggest that the presence of a greater number of hydrogen-bond donors on/around A-ring improves the leishmanicidal activity of this kind of compound. A similar pattern was observed by Torres-Santos et al. who evaluated the anti-leishmanial effect (*L. amazonensis*, MPRO/BR/72/M 1841 strain LV79) of different ursane- and oleanane-type triterpenes and found that 3β hydroxylation of oleanolic acid, tormentic acid, and 2α,3β-dihydroxyolean-12-en-28-oic acid importantly accounts for their leishmanicidal activity, while the presence of hydroxyl groups at positions 2α and 19 is not determinant for bioactivity [62]. Another study, carried out with 2α,3β-dihydroxy-oleanane-type triterpenes, showed that maslinic acid displayed better activity than oleanolic acid against a *L. amazonensis* strain (MHOM/BR/77LTB0016); however, the latter was more potent than oleanolic acid on *L. infantum* amastigotes (MHOM/ES/1996/BCN-143) [63]. Moreover, Gnoatto et al. demonstrated that 3β acetylation of oleanolic acid increased about fivefold its leishmanicidal activity against *L. amazonensis* (MHOM/BR/1987/BA), but not against *L. infantum* (MCAN/GR/82/LEM497) [64]. These results evidence that the anti-leishmanial effect of pentacyclic triterpenes of the oleanane group is dependent on the parasite species and strains. Not only the presence, but also the stereochemistry of the hydroxyl group at C-3 greatly influences the effect on *Leishmania*, since it was demonstrated that oleanolic acid (IC_50_ = 91 nM) is approximately 200-fold more potent than its corresponding isomer, *epi*-oleanolic acid (IC_50_ = 18.8 μM), against *L. donovani* promastigotes [65,66], which suggests the interaction of these compounds with a biological target.

The investigations that were carried out in order to elucidate the mechanism of action of anti-*Leishmania* molecules indicate that they act on targets that participate in metabolic pathways essential for the survival of this parasite, such as glyoxalase, trypanothion, dihydrofolate reductase (DHFR), topoisomerase, metacaspases, MAP kinases, polyamines, purine, glycolytic, and sterol pathways [22,67]. Some of the natural products and synthetic compounds that were shown to interact with those targets include flavones (glyoxalase), trivalent antimonials, azoles, and isoquinoline alkaloids (trypanothion), pentavalent antimonials and lupane (topoisomerase I and IB, respectively), staurosporine (MAP kinases), allopurinol and iminorbitol (purines), and statins, bisphosphonate, azasterols, and azoles (sterol biosynthesis) [67,68,69].

Concerning oleanane-type triterpenes, there is currently no information on their mechanism of action. However, in a study carried out with oleanolic acid, Torres-Santos et al. [62] found that this compound did not induce macrophage NO production. Later, Souza et al. confirmed this result when demonstrating that oleanolic acid, which possessed a good activity against *L. amazonensis*, *L. braziliensis*, and *L. infantum* (IC_50_ < 70 μM in promastigotes and amastigotes), did not provoke FN-γ–assisted macrophage activation [70]. These findings indicated that this type of triterpene most likely acts on targets located in *Leishmania* parasites rather than in macrophages. Additionally, Sousa et al. carried out molecular docking experiments to explore whether oleanolic acid was capable of binding to sterol 14α-demethylase cytochrome P450 (CYP51), a necessary enzyme for sterol biosynthesis. They found that this triterpene interacts with a hydrophobic pocket of CYP51 in a similar manner to lanosterol, the natural substrate. However, unlike lanosterol whose methyl at position C-14 is oriented toward the Fe^2+^ atom of the heme group, in the case of oleanolic acid, it is the C-28 carboxyl group that is positioned near the heme group [70].

Lantadene B (**10**), lantanilic acid (**8**), and camaric acid (**9**), isolated from the pink-yellow cultivar of *L. camara*, have the same oleanane core skeleton as oleanolic acid, but they possess acyl moieties instead of a carboxyl group. Considering the structural similarities between oleanolic acid and oleanane-type triterpenes from *L. camara*, it could be hypothesized that these compounds are capable of binding to CYP51. Moreover, taking into account that the triterpenes from *L. camara* display a more potent leishmanicidal activity (IC_50_ < 25 μM) than oleanolic acid, it is possible to assume that this difference in potency is due to the presence of the acyl functionalities. In fact, a molecular docking analysis of sterol-type compounds, including *epi*-oleanolic acid, and other oleananes showed that modifications in the functional groups of the oleanane core greatly influence the position of the molecules inside the hydrophobic pocket of CYP51, which affects their interaction with the heme group [71]. Conducting an in silico study, similar to those previously reported, would certainly contribute to a better understanding of the mechanism of action of the triterpenes we obtained from the Mexican cultivar of *L. camara*.

Another potential mechanism of action underlying the leishmanicidal effect of oleanane-type triterpenes might be related to their structural similarity to ergosterol, which is an important constituent of lipid rafts of the *Leishmania* membrane [72,73]. Goad and collaborators were the first to identify the major sterols in the cellular membrane of nine *Leishmania* strains, and found that ergosta-5,7,24(28)-trien-3β-ol was the most abundant, whereas cholesterol was found at trace concentrations, as *Leishmania* species do not possess the enzymatic machinery to synthesize this sterol [72]. When these researchers cultured a *L. tropica* strain in a culture medium enriched with cholesterol, the membrane lipid profile was modified and showed significant cholesterol levels (33.5%), while other sterols diminished considerably, evidencing that *Leishmania* was able to incorporate extracellular cholesterol into its membrane [72]. Therefore, as lantanilic (**8**) and camaric (**9**) acids and lantadenes A and B (**10**) are very similar to the cholesterol molecule, it is possible that these triterpenes might be embedded into *Leishmania* membrane. On the other hand, considering that ergosterols are mainly located in the lipid rafts, it is possible that oleanane triterpenes could be incorporated into these membrane microdomains, positioning the polar groups (located in or around C-3 of A-ring) in the same direction as the polar heads of sphingolipids, which causes membrane pore formation, thus provoking an osmotic imbalance that leads to *Leishmania* death. Nevertheless, this hypothesis needs to be confirmed.

In addition to their leishmanicidal effect, other biological activities were demonstrated for the oleanane-type triterpenes obtained from the pink-yellow cultivar of *L. camara*. Lantadene B (**10**) possesses antiviral, antitumoral, allelopathic, and antibacterial properties [30], while both lantanilic (**8**) and camaric acids (**9**) displayed nematicidal [74,75], antibacterial, and antitubercular activities [76]. In addition, camaric acid inhibited casein kinase II enzymatic activity [77] and showed mosquito larvicidal [78] and antiprotozoal effects [79].

Some studies evidenced that lantadene B (**10**) induces hepatotoxicity, nefrotoxicity, and photosensitization on guinea pigs, cattle, and sheep [80]; however, this triterpene was not toxic for rats and rabbits. This behavior was attributable to the microbiota of these rodents [30,81]. On the other hand, there are no reports about the toxicity of lantanilic (**8**) and camaric (**9**) acids. Evidently, it is necessary to carry out in vitro and in vivo toxicological studies to determine the safety of these compounds. Oleanane-type triterpenes have an adequate lipophilicity, which allows them to permeate skin. Therefore, these compounds could be employed to prepare a topical formulation to treat cutaneous leishmaniasis, in the case that their systemic toxicity represents a risk. It is also possible to develop a topical phytomedicine based on a triterpene-enriched standardized extract of *L. camara*.

The results obtained in the present study contribute to support the potential of this pink-yellow cultivar of *L. camara* for the development of an anti-leishmanial phytopharmaceutical. Our findings also allow us to propose lantadene B (**10**), lantanilic acid (**8**), and camaric acid (**9**) as valuable lead compounds to be developed as drugs for the treatment of leishmaniasis.

## 3. Materials and Methods

### 3.1. Chemicals

Dichloromethane, hexane, ethyl acetate, acetonitrile, and methanol were purchased from JT Baker, Mexico. Silica gel 60 Å, 70–230 mesh, 63–200 μm; Silica gel thin-layer chromatography (TLC) aluminum foils 20 × 20 cm, 200 μm thickness, 8.0–12.0 μm particle size, 60 Å pore diameter; Roswell Park Memorial Institute (RPMI-1640) medium, Schneider medium, fetal bovine serum (FBS), trifluoroacetic acid (TFA), eicosane (**1**), squalene (**2**), β-ionone (**3**), caryophyllene oxide (**4**), β-caryophyllene (**5**), hexanoic acid (**6**), and trans-2-methyl-2-butenoic acid (tiglic acid, **7**) were purchased from Sigma-Aldrich. M199 medium and Grace’s medium were purchased from Gibco (Life Technologies).

### 3.2. Biological Material

Axenic promastigotes and amastigotes of *Leishmania mexicana* originally obtained from lesions of a patient with localized cutaneous leishmaniasis (LCL) from Quintana Roo, Mexico, while intracellular amastigotes of *Leishmania amazonensis* strain MHOM/BR/77/LTB0016 were used. *L. amazonensis* (MHOM/BR/77/LTB0016) was kindly provided by the Department of Immunology, Oswaldo Cruz Foundation (FIOCRUZ), Brazil. Peritoneal macrophages were obtained from 20–22-g female BALB/c mice, which were obtained from The National Center of Laboratory Animals Production (CENPALAB, Cuba). Protocol for animal use and handling was evaluated and approved by the Ethics Committee of the Institute of Tropical Medicine “Pedro Kouri”, Havana, Cuba (CEI-IPK14–12).

### 3.3. Plant Material

Aerial parts (leaves, stems, flowers, and fruits) of a pink-yellow cultivar of *Lantana camara* var. *aculeata* (L.) were collected in March and April 2014 at the gardens of Universidad Autónoma de Querétaro, Cerro de las Campanas, Querétaro, Querétaro (20°35’25.4” N, 100°24’38.3” W). *L. camara* was previously identified by Dr. Heike Vibrans. A specimen was deposited at the National Herbarium of Mexico (MEXU), No. Voucher 1414005.

### 3.4. Extraction and Isolation

The plant was dried at room temperature protected from light for two weeks. After drying, 6.5 kg of aerial parts of *L. camara* were hand-fragmented and then ground in an electric mill with a 0.5-mm-diameter mesh. The obtained powder was macerated with dichloromethane for a week. Vegetal material was filtered and extracted once again with fresh solvent for another week. Both extracts were pooled and concentrated at reduced pressure. Then, 122 g of dried crude extract was subjected to column chromatography, using silica gel 60 Å, 70–230 mesh, 63–200 μm as the stationary phase. The size of the column was 10 cm in diameter × 70 cm in height. Hexane and ethyl acetate mixtures in different proportions (100:0, 98:2, 95:5, 9:1, 8:2, 7:3, 6:4, 1:1, and 0:100) were used as the mobile phase; finally, most polar components were eluted with methanol. In total, 325 fractions were collected in 250-mL volumes and pooled in 18 final fractions (FI–FXVIII) according to their chromatographic similarity in TLC (stationary phase: silica gel 60; mobile phase: hexane and ethyl acetate, 3:2), detecting the spots under ultraviolet (UV) light (254 and 365 nm), followed by spraying of Liebermann–Burchard’s reagent and heating at 110 °C for 2 min. Fractions were concentrated at reduced pressure and the organic residues were stored in refrigeration (4 °C) until their use in the leishmanicidal and cytotoxic bioassays. Fraction II (FII, 400 mg, yellow waxy mass with floral odor) was obtained by the elution of the open column chromatography (OCC) with hexane 100%. Fraction II was analyzed using GC–MS for the identification of its main components. Fractions FX (2.5 g, green powder) and FXI (2.25 g, green powder) were obtained with the hexane–ethyl acetate (8:2) elution system. Both fractions were separately washed with hexane–methanol–water (1:1:1) to remove chlorophylls. Greenish precipitates were formed in the aqueous phase. The precipitate of FXI was vacuum-filtered and washed with cold methanol yielding an amorphous yellowish powder (liqueur) and some greenish needle-type crystals (filtered). The amorphous powder was vaccum-filtered once again, while the needles were washed with cold hexane, until the removal of the color. Then, 28 mg of beige needles were obtained and dried for their characterization by NMR. Fraction XV (4 g, dark green powder, acrid odor) was washed with cold ethyl acetate and yielded a white amorphous powder. Chlorophyll-free fractions FX, FXI, and FXV were chromatographically analyzed to determine if they contained common components.

### 3.5. GC–MS Analysis of FII

For the identification of the major components of fraction II, an Agilent Gas Chromatographer 7890A series (Agilent Technologies, Inc., Santa Clara, CA, USA) was used. The injection port and detector temperature was 230 °C. Split (20:1) mode was used for injection. A capillary column HP-5MS 60 m × 0.25 mm inner diameter (i.d.), 0.25 μm thickness (Agilent Technologies, Inc.) was used for the analysis. The oven temperature was programmed as follows: initial temperature of 40 °C for 5 min; then, temperature was risen at a rate of 5 °C/min until 230 °C and maintained for 15 min. As a carrying gas, helium was used at a constant flow of 1 mL/min. The relative abundance of each compound was determined from its area under the curve. Mass spectrometry was achieved using an electron impact mass spectrometer Agilent 5975C series with a quadrupole analyzer. The temperatures of the ion source and the quadrupole were 230 and 250 °C, respectively. The transfer line was set at 280 °C. A complete screening was used in a rank 33 to 300 amu, with a scanning rate of 5.2 s^−1^ and an ionization voltage of 70 eV. The MSD Chemstation E.0100237 software was employed for data analysis. Compound identification was performed by comparing spectral data with data from the NIST/EPA/NIH Mass Spectra Library, version 1.7, USA.

### 3.6. HPLC-PDA Analysis of Fractions FX, FXI, and FXV

A Waters™ HPLC-PDA (pump 600, controller 600S, and PDA detector 2998) was used for the chromatographic analysis of fractions FX, FXI, and, FXV. The HPLC conditions were as follows: reverse stationary phase (Waters™ RP-18 Xterra Shield, 125 Å, 3.5 µm, 4.6 × 150 mm); mobile phase: 70% acetonitrile and 30% acidulated water (0.1% TFA); flux: 1 mL/min, λ = 210 nm.

### 3.7. NMR Analysis for the Identification of the Triterpenes Isolated from Fractions FX, FXI, and FXV

An Avance III HD 700 spectrometer operating at an ^1^H frequency of 699.95 MHz (Bruker, Billerica, MA, USA) equipped with a 5-mm *z*-axis gradient TCI cryoprobe was used for the acquisition of the NMR spectra of the triterpenes isolated from fractions FX, FXI, and FXV: lantadene B (**10**), lantanilic acid (**8**), and camaric acid (**9**). NMR experiments were recorded using standard Bruker pulse sequences in 5-mm NMR tubes at 298 K. The chemical shifts are reported in ppm relative to the solvent resonance as the internal standard (CDCl_3_). Coupling constants (*J*) are given in Hz. For detailed NMR conditions, see Delgado-Altamirano et al. (2019) [55].

### 3.8. In Vitro Biological Testing

Stock solutions of the whole crude extract and the chromatographic fractions were prepared at a concentration of 20 mg/mL by dilution in dimethyl sulfoxide (DMSO) for the evaluation of the cytotoxic and leishmanicidal activity on amastigotes of *L. amazonensis* and promastigotes of *L. mexicana*. For the analysis of the leishmanicidal activity on amastigotes of *L. mexicana*, fractions were diluted in absolute ethanol On the other hand, stock solutions of the compounds identified in the bioactive fractions were diluted in DMSO at a concentration of 0.05 M. Pentamidine (Richet, Buenos Aires, Argentina) was used as a reference drug for *L. amazonensis*, while Glucantime® (Sanofi-Aventis, São Paulo, Brazil) was used for *L. mexicana*. Stock solutions were kept at −20 °C until their biological evaluation.

#### 3.8.1. Cytotoxicity Assay on BALB/c Mice Peritoneal Macrophages

BALB/c mice were euthanized by cervical dislocation and peritoneal macrophages were extracted by washing with RPMI-1640 medium. Then, 100 μL of the cellular suspension (1–3 × 10^6^ cells/mL) was distributed in 96-well plates, before being incubated for 2 h at 37 °C and 5% CO_2_. To macrophage monolayers, we added 100 μL of RPMI-1640 medium supplemented with antibiotics (200 IU penicillin and 200 μg/mL streptomycin) and different concentrations of DMSO-dissolved fractions to obtain a final concentration between 12.5 and 200 μg/mL. In each assay, there was a control with non-treated macrophages. The plates were incubated for 48 h; then, 15 μL of MTT (5 mg/mL in SS) was added and the plate was incubated. After 4 h, the supernatant was removed and the tetrazolium precipitate was dissolved in 100 μL of DMSO. Absorbance was measured in a spectrophotometer at 560 nm and at 630 nm as the reference wavelength [27,54].

#### 3.8.2. In Vitro Anti-Amastigote Activity on *Leishmania amazonensis*

Peritoneal macrophages of BALB/c mice were extracted in the same conditions as in the cytotoxicity assay. They were distributed in a 24-well plate with 1 mL of RPMI-1640 in each well. Macrophages were incubated at 37 °C, 5% CO_2_ for 2 h. Non-adherent cells were removed. Adherent macrophages were infected with stationary promastigotes of *L. amazonensis* in a ratio of four parasites to each macrophage. The plate was incubated in the same previous conditions for 4 h. Then, free promastigotes were removed, and 1990 μL of fresh medium RPMI-1640 supplemented with antibiotics and 10% HI-FBS was added. For the different groups, 10 μL of the fractions or DMSO was added to obtain the concentrations 6.25, 12.5, 25, 50, 100, and 200 μg/mL. As a positive control, pentamidine (10 mg/mL) was used. The cultures were incubated for 48 h at 37 °C and 5% CO_2_. Afterward, the monolayer was fixed with MeOH and stained with Giemsa. The bottom of the wells was analyzed in a zig-zag pattern with an optical microscope, using an oil immersion objective to count the number of amastigotes in 25 macrophages, so as to determine the percentage of infected macrophages [27,54].

#### 3.8.3. In Vitro Anti-Promastigote Activity on *Leishmania mexicana*

Axenic late log-phase parasites were used. In 5-mL Falcon® tubes, we prepared the following concentrations from the crude extract and the chromatographic fractions: 6.25, 12.5, 25, 50, 100, and 200 μg/mL. For the pure compounds, the concentrations prepared were 1, 10, 50, 100, 200, and 500 μM. To obtain these concentrations, the dilutions were prepared from their respective stock solution into 2 mL of medium 199 supplemented with 10% heat-inactivated FBS. Then, 1 × 10^6^ parasites/mL were added. The tubes where incubated for 72 h at 26 °C. After this time, the parasites were fixed with glutaraldehyde 1% and their number was established by counting on a Neubauer chamber. DMSO was used as the negative control, while Glucantime was used as the positive control.

#### 3.8.4. In Vitro Anti-Amastigote Activity on *Leishmania mexicana*

Axenic rate log-phase parasites were used. In 1.5-mL microcentrifuge tubes, we prepared the following concentrations from the crude extract and the chromatographic fractions: 12.5, 25, 50, 100, and 200 μg/mL. To obtain these concentrations, the dilutions were prepared from their respective stock solution into 1 mL of Grace’s medium supplemented with 20% heat-inactivated FBS. Then, 1 × 10^6^ parasites/mL were added. The tubes where incubated for 72 h at 33 °C. After this time, the parasites were fixed with glutaraldehyde 1% and their number was established by counting on a Neubauer chamber. For this assay, absolute alcohol was used as the negative control, while Glucantime was used as the positive control.

#### 3.8.5. Selectivity Index

This is defined as the quotient of the half maximal cytotoxic concentration (CC_50_) for macrophages divided by the IC_50_ for the parasites.

### 3.9. Statistical Analysis

Anti-leishmanial activity and cytotoxicity assays were performed by triplicate. The values of IC_50_ and CC_50_ were established from a non-linear regression of dose–response curves in GraphPad Prism 6.01 Software, Inc. All results are expressed as their average and standard deviation. One- and two-way ANOVA analyses with a Tukey’s post hoc test were performed for comparing the IC_50_ values of pure compounds (*p* < 0.0001) and fractions (*p* < 0.05), respectively.

## 4. Conclusions

The present study demonstrated that the dichloromethane extract obtained from a Mexican pink-yellow cultivar of *L. camara* contains secondary metabolites that possess in vitro anti-leishmanial activity against *L. mexicana* promastigotes. The anti-leishmanial activity of eicosane (**1**), squalene (**2**), β-ionone (**3**), hexanoic acid (**6**), tiglic acid (**7**), camaric acid (**9**), and lantadene B (**10**) was reported for the first time. Lantadene B (**10**) and a mixture consisting of 79% lantanilic acid (**8**) and 21% camaric acid (**9**) were the most active. This study indicates that this cultivar of *L. camara* has great potential for the development of phytopharmaceuticals or as a source of natural product lead compounds for the design of new drugs to treat leishmaniasis.

## Figures and Tables

**Table 1:** Half maximal cytotoxic and inhibitory concentration (CC_50_ and IC_50_) values for the crude extract and the 18 chromatographic fractions from *Lantana camara*.

Extract/Fraction	CC_50_ ± SD (μg/mL) BALB/c Peritoneal Macrophages	IC_50_ ± SD (μg/mL) *Leishmania amazonensis* Amastigotes	SI on *L. amazonensis*	IC_50_ ± SD (μg/mL) *L. mexicana* Promastigotes	SI on *L. mexicana*
Crude extract	>100	21.8 ± 2.4	>5	42.6 ± 1.9	>2
FI	65.2 ± 2.3	12.2 ± 1.8	5	>200	<0.5
FII	>100	**9.1 ± 3.4**	**>11**	132.1 ± 7.9	0.49
FIII	>100	18.3 ± 1.5	>5	>200	N.C.
FIV	>100	30.2 ± 3.5	>3	>200	<0.5
FV	>100	17.9 ± 3.8	>6	>200	<0.5
FVI	>100	21.5 ± 4.3	>5	>200	<0.5
FVII	96.8 ± 2.2	17.9 ± 2.5	>5	>200	<0.5
FVIII	92.3 ± 3.1	24.7 ± 2.9	>4	>200	<0.5
FIX	>100	18.6 ± 1.5	>5	96.0 ± 1.6	>1
FX	>100	**7.9 ± 0.3**	**>13**	**11.2 ± 2.2**	**>9**
FXI	>100	**8.0 ± 1.1**	**>13**	**22.0 ± 9.3**	**>4**
FXII	>100	16.7 ± 3.7	>6	87.3 ± 2.1	>1
FXIII	82.2 ± 2.6	12.1 ± 0.7	>7	83.3 ± 6.9	1
FXIV	60.8 ± 1.4	15.1 ± 1.0	>4	89.4 ± 2.4	N.C.
FXV	>100	23.6 ± 2.6	>4	**2.8 ± 0.5**	**>35**
FXVI	>100	**8.5 ± 1.7**	**>12**	**16.5 ± 1.6**	**>6**
FXVII	>100	19.8 ± 3.9	>5	157.8 ± 15.5	<0.6
FXVIII	>100	15.6 ± 4.7	>6	>200	<0.5
Pentamidine	11.7 ± 1.7	1.3 ± 0.1	9	N.D.	N.D.
Glucantime®	60.0 ± 3.0	N.D.	N.D.	4019.7 ± 140.4	0.015

SI: selectivity index; N.C.: not calculated; N.D.: not determined. Two-way ANOVA analysis with Tukey’s post-hoc test, *p* < 0.05. Bold letters highlight the most active fractions.

**Table 2:** IC_50_ values for the leishmanicidal activity elicited by the pure compounds identified in the most active fractions.

Fraction	Compound	IC_50_ ± SD (μM) *L. mexicana* promastigotes
FII	Eicosane (**1**)	>500 ^a^
	Squalene (**2**)	>500 ^a^
	β-ionone (**3**)	80.80 ± 7.09 ^b^
	Caryophyllene oxide (**4**)	81.62 ± 2.16 ^b^
	β-caryophyllene (**5**)	74.43 ± 4.38 ^b^
	Hexanoic acid (**6**)	42.96 ± 6.02 ^c^
	Tiglic acid (**7**)	>500 ^a^
FX, FXV	Lantanilic acid (**8**)/Camaric acid (**9**) (79%/21%)	12.02 ± 0.36 ^d^ (9.50 ± 0.28 lantanilic acid, **8**) (2.52 ± 0.08 camaric acid, **9**)
FX, FXI	Lantadene B (**10**)	23.45 ± 2.15 ^d^
	Glucantime®	11,000.00 ± 2.15 ^e^

Superindexed letters (a, b, c, d and e) indicate the significant statistical differences between the IC_50_ of the pure compounds after the one-way ANOVA analysis with Tukey’s post hoc test, *p* < 0.0001.

## Supplementary Materials

Supplementary material can be found at http://www.mdpi.com/1422-0067/20/4/872/s1.

## References

[1] Alvar J., Vélez I.D., Bern C., Herrero M., Desjeux P., Cano J., Jannin J., de Boer M. (2012). Leishmaniasis worldwide and global estimates of its incidence. PLoS ONE.

[2] Akhoundi M., Kuhls K., Cannet A., Votýpka J., Marty P., Delaunay P., Sereno D. (2016). A historical overview of the classification, evolution, and dispersion of *Leishmania* parasites and sandflies. PLoS Negl. Trop. Dis..

[3] Rodrigues V., Cordeiro-da-Silva A., Laforge M., Silvestre R., Estaquier J. (2016). Regulation of immunity during visceral *Leishmania* infection. Parasit. Vectors.

[4] Scott P., Novais F.O. (2016). Cutaneous leishmaniasis: Immune responses in protection and pathogenesis. Nat. Rev. Immunol..

[5] Séguin O., Descoteaux A. (2016). *Leishmania*, the phagosome, and host responses: The journey of a parasite. Cell. Immunol..

[6] Ibrahim M.K., Barnes J.L., Anstead G.M., Jimenez F., Travi B.L., Peniche A.G., Osorio E.Y., Ahuja S.S., Melby P.C. (2013). The malnutrition-related increase in early visceralization of *Leishmania donovani* is associated with a reduced number of lymph node phagocytes and altered conduit system flow. PLoS Negl. Trop. Dis..

[7] Ibrahim M.K., Barnes J.L., Osorio E.Y., Anstead G.M., Jimenez F., Osterholzer J.J., Travi B.L., Ahuja S.S., White A.C., Melby P.C. (2014). Deficiency of lymph node-resident dendritic cells (DCs) and dysregulation of DC chemoattractants in a malnourished mouse model of *Leishmania donovani* infection. Infect. Immun..

[8] Malafaia G. (2009). Protein-energy malnutrition as a risk factor for visceral leishmaniasis: A review. Parasite Immunol..

[9] Kumar V., Bimal S., Singh S.K., Chaudhary R., Das S., Lal C., Pandey K., Das V.R., Das P. (2014). *Leishmania donovani*: Dynamics of *L. donovani* evasion of innate immune cell attack due to malnutrition in visceral leishmaniasis. Nutrition.

[10] Lestinova T., Rohousova I., Sima M., De Oliveira C.I., Volf P. (2017). Insights into the sand fly saliva: Blood-feeding and immune interactions between sand flies, hosts, and *Leishmania*. PLoS Negl. Trop. Dis..

[11] Reithinger R., Dujardin J., Louzir H., Pirmez C., Alexander B., Brooker S. (2007). Cutaneous leishmaniasis. Lancet Infect. Dis..

[12] Pedrosa M.S.C. (2017). Clinical manifestations of visceral leishmaniasis (American visceral leishmaniasis). The Epidemiology and Ecology of Leishmaniasis.

[13] Özkeklikçi A., Karakuş M., Özbel Y., Töz S. (2017). The new situation of cutaneous leishmaniasis after Syrian civil war in Gaziantep city, Southeastern region of Turkey. Acta Trop..

[14] Doganay M., Demiraslan H. (2016). Refugees of the Syrian civil war: Impact on reemerging infections, health services, and biosecurity in Turkey. Heal. Secur..

[15] Desjeux P. (2004). Leishmaniasis: Current situation and new perspectives. Comp. Immunol. Microbiol. Infect. Dis..

[16] Desjeux P. (2001). Worldwide increasing risk factors for leishmaniasis. Med. Microbiol. Immunol..

[17] Oryan A., Akbari M. (2016). Asian Paci fi c Journal of Tropical Medicine. Asian Pac. J. Trop. Med..

[18] Alasaad S. (2013). War diseases revealed by the social media: Massive leishmaniasis outbreak in the Syrian spring. Parasit. Vectors.

[19] Kumar A.H., Sen P., Roy S. (2011). Use of antimony in the treatment of leishmaniasis: Current status and future directions. Mol. Biol. Int..

[20] Sundar S., Chakravarty J. (2010). Antimony toxicity. Int. J. Environ. Res. Public Health.

[21] Rajasekaran R., Chen Y.-P.P. (2015). Potential therapeutic targets and the role of technology in developing novel antileishmanial drugs. Drug Discov. Today.

[22] Singh N., Mishra B.B., Bajpai S., Singh R.K., Tiwari V.K. (2014). Natural product based leads to fight against leishmaniasis. Bioorg. Med. Chem..

[23] Corpas-López V., Morillas-Márquez F., Navarro-Moll M.C., Merino-Espinosa G., Díaz-Sáez V., Martín-Sánchez J. (2015). (−)-α-bisabolol, a promising oral compound for the treatment of visceral leishmaniasis. J. Nat. Prod..

[24] Corpas-López V., Merino-Espinosa G., López-Viota M., Gijón-Robles P., Morillas-Mancilla M.J., López-Viota J., Díaz-Sáez V., Morillas-Márquez F., Navarro Moll M.C., Martín-Sánchez J. (2016). Topical treatment of *Leishmania tropica* infection using (−)-α-bisabolol ointment in a hamster model: Effectiveness and safety assessment. J. Nat. Prod..

[25] Kyriazis J.D., Aligiannis N., Polychronopoulos P., Skaltsounis A.L., Dotsika E. (2013). Leishmanicidal activity assessment of olive tree extracts. Phytomedicine.

[26] Kyriazis I.D., Koutsoni O.S., Aligiannis N., Karampetsou K., Skaltsounis A.-L., Dotsika E. (2016). The leishmanicidal activity of oleuropein is selectively regulated through inflammation- and oxidative stress-related genes. Parasit. Vectors.

[27] Delgado-Altamirano R., Monzote L., Piñón-Tápanes A., Vibrans H., Rivero-Cruz J.F., Ibarra-Alvarado C., Rojas-Molina A. (2017). In vitro antileishmanial activity of Mexican medicinal plants. Heliyon.

[28] Ghisalberti E.L. (2000). *Lantana camara* L. (Verbenaceae). Fitoterapia.

[29] Goncalves E., Herrera I., Duarte M., Bustamante R.O., Lampo M., Velásquez G., Sharma G.P., García-Rangel S. (2014). Global invasion of *Lantana camara*: Has the climatic niche been conserved across continents?. PLoS ONE.

[30] Sharma O.P., Sharma S., Pattabhi V., Mahato S.B., Sharma P.D. (2007). A review of the hepatotoxic plant *Lantana camara*. Crit. Rev. Toxicol..

[31] Bhagwat S.A., Breman E., Thekaekara T., Thornton T.F., Willis K.J. (2012). A battle lost? Report on two centuries of invasion and management of *Lantana camara* L. in Australia, India and South Africa. PLoS One.

[32] Gonçalves Ferreira Macedo J., Alencar De Menezes I.R., Alves Ribeiro D., De Oliveira Santos M., Gonçalves De Mâcedo D., Ferreira Macêdo M.J., Vilar De Almeida B., Geraldo Souza De Oliveira L., Pereira Leite C., De Almeida Souza M.M. (2018). Analysis of the variability of therapeutic indications of medicinal species in the Northeast of Brazil: Comparative study. Evid.-Based Complement. Altern. Med..

[33] Bisi-Johnson M.A., Obi C.L., Hattori T., Oshima Y., Li S., Kambizi L., Eloff J.N., Vasaikar S.D. (2011). Evaluation of the antibacterial and anticancer activities of some South African medicinal plants. BMC Complement. Altern. Med..

[34] Cheruiyot K.R., Olila D., Kateregga J. (2009). In-vitro antibacterial activity of selected medicinal plants from Longisa region of Bomet district, Kenya. Afr. Health Sci..

[35] Moyo B., Masika P.J., Dube S., Maphosa V. (2009). An *in-vivo* study of the efficacy and safety of ethno-veterinary remedies used to control cattle ticks by rural farmers in the Eastern Cape Province of South Africa. Trop. Anim. Health Prod..

[36] Jonville M.C., Kodja H., Humeau L., Fournel J., De Mol P., Cao M., Angenot L., Frédérich M. (2008). Screening of medicinal plants from Reunion Island for antimalarial and cytotoxic activity. J. Ethnopharmacol..

[37] Magassouba F.B., Diallo A., Kouyaté M., Mara F., Mara O., Bangoura O., Camara A., Traoré S., Diallo A.K., Zaoro M. (2007). Ethnobotanical survey and antibacterial activity of some plants used in Guinean traditional medicine. J. Ethnopharmacol..

[38] Dabur R., Gupta A., Mandal T.K., Singh D.D., Bajpai V., Gurav A.M., Lavekar G.S. (2007). Antimicrobial activity of some Indian medicinal plants. Afr. J. Tradit. Complement. Altern. Med..

[39] Basu S., Hazra B. (2006). Evaluation of nitric oxide scavenging activity, in vitro and ex vivo, of selected medicinal plants traditionally used in infammatory diseases. Phyther. Res..

[40] Hernández T., Canales M., Avila J.G., Duran A., Caballero J., Romo de Vivar A., Lira R. (2003). Ethnobotany and antibacterial activity of some plants used in traditional medicine of Zapotitlán de las Salinas, Puebla (México). J. Ethnopharmacol..

[41] Begum S., Ayub A., Zehra S.Q., Siddiqui B.S., Choudhary M.I., Sciences B. (2014). Leishmanicidal triterpenes from *Lantana camara*. Chem. Biodivers..

[42] Braga F.G., Bouzada M.L.M., Fabri R.L., Matos M.D.O., Moreira F.O., Scio E., Coimbra E.S. (2007). Antileishmanial and antifungal activity of plants used in traditional medicine in Brazil. J. Ethnopharmacol..

[43] Weyerstahl P., Marschall H., Eckhardt A., Christiansen C. (1999). Constituents of commercial Brazilian lantana oil. Flavour Fragr. J..

[44] Santos I.E.M. (2002). A taxonomic revision of *Lantana* sect. *Lantana* (Verbenaceae) in the Greater Antilles. Willdenowia.

[45] Queensland D. (2003). Lantana—A Weed of National Significance Identification guide: *Lantana* Flowers. https://www.daf.qld.gov.au/__data/assets/pdf_file/0004/60376/IPA-Lantana-Flowers-Id-Guide.pdf.

[46] Sharma O.P., Vaid J., Sharma P.D. (1991). Comparison of lantadenes content and toxicity of different taxa of the *Lantana* plant. J. Chem. Ecol..

[47] Randrianalijaona J.-A., Ramanoelina P.A.R., Rasoarahona J.R.E., Gaydou E.M. (2005). Seasonal and chemotype influences on the chemical composition of *Lantana camara* L.. Anal. Chim. Acta.

[48] Ghobakhloo N., Motazedian M.H., Pourmohammadi B., Yousefi Z. (2017). Evaluation of correlation between the in vitro susceptibility of field isolates of *Leishmania major* and clinical outcomes of meglumine antimoniate therapy in Fars Province, Iran. J. Arthropod. Borne. Dis..

[49] Perez-Franco J.E., Cruz-Barrera M.L., Robayo M.L., Lopez M.C., Daza C.D., Bedoya A., Mariño M.L., Saavedra C.H., Echeverry M.C. (2016). Clinical and parasitological features of patients with American cutaneous leishmaniasis that did not respond to treatment with meglumine antimoniate. PLoS Negl. Trop. Dis..

[50] Martín-Montes Á., Plano D., Martín-Escolano R., Alcolea V., Díaz M., Pérez-Silanes S., Espuelas S., Moreno E., Marín C., Gutiérrez-Sánchez R. (2017). Library of seleno-compounds as novel agents against *Leishmania* species. Antimicrob. Agents Chemother..

[51] Mahmoudvand H., Kheirandish F., Mirbadie S.R., Kayedi M.H., Rezaei Riabi T., Ghasemi A.A., Bamorovat M., Sharifi I. (2017). The potential use of methotrexate in the treatment of cutaneous leishmaniasis: *In vitro* assays against sensitive and meglumine antimoniate-resistant strains of *Leishmania tropica*. Iran J. Parasitol..

[52] Ginouvès M., Simon S., Nacher M., Demar M., Carme B., Couppié P., Prévot G. (2017). In vitro sensitivity of cutaneous *Leishmania* promastigote isolates circulating in French guiana to a set of drugs. Am. J. Trop. Med. Hyg..

[53] Cos P., Vlietinck A.J., Vanden D., Maes L. (2006). Anti-infective potential of natural products: How to develop a stronger in vitro ‘proof-of-concept’. J. Ethnopharmacol..

[54] García M., Monzote L., Scull R., Herrera P. (2012). Activity of Cuban plants extracts against *Leishmania amazonensis*. ISRN Pharmacol..

[55] Delgado-Altamirano R., Rojas A., Esturau-Escofet N. (2019). 1H and 13C NMR reassignment of some chemical shifts of lantanilic acid and camaric acid. Magn. Reson. Chem..

[56] Monzote L., García M., Pastor J., Gil L., Scull R., Maes L., Cos P., Gille L. (2014). Essential oil from *Chenopodium ambrosioides* and main components: Activity against *Leishmania*, their mitochondria and other microorganisms. Exp. Parasitol..

[57] Monzote L., Geroldinger G., Tonner M., Scull R., De Sarkar S., Bergmann S., Bacher M., Staniek K., Chatterjee M., Rosenau T. (2018). Interaction of ascaridole, carvacrol, and caryophyllene oxide from essential oil of *Chenopodium ambrosioides* L. with mitochondria in *Leishmania* and other eukaryotes. Phyther. Res..

[58] Monzote L., Pastor J., Scull R., Gille L. (2014). Antileishmanial activity of essential oil from *Chenopodium ambrosioides* and its main components against experimental cutaneous leishmaniasis in BALB/c mice. Phytomedicine.

[59] DNDi Drugs for Neglected Diseases iniciative: Leishmaniasis. https://www.dndi.org/diseases-projects/leishmaniasis/.

[60] Soares D.C., Portella N.A., Ramos M.F.D.S., Siani A.C., Saraiva E.M. (2013). Trans-β-Caryophyllene: An effective antileishmanial compound found in commercial copaiba Oil (*Copaifera* spp.). Evid.-Based Complement. Altern. Med..

[61] Ridoux O., Di Giorgio C., Delmas F., Elias R., Mshvildadze V., Dekanosidze G., Kemertelidze E., Balansard G., Timon-David P. (2001). In vitro antileishmanial activity of three saponins isolated from ivy, α-hederin, β-hederin and hederacolchiside A1, in association with pentamidine and amphotericin B. Phyther. Res..

[62] Torres-Santos E.C., Lopes D., Rodrigues Oliveira R., Carauta J.P.P., Bandeira Falcao C.A., Kaplan M.A.C., Rossi-Bergmann B. (2004). Antileishmanial activity of isolated triterpenoids from *Pourouma guianensis*. Phytomedicine.

[63] Sifaoui I., López-Arencibia A., Martín-Navarro C.M., Reyes-Batlle M., Mejri M., Lorenzo-Morales J., Abderabba M., Piñero J.E. (2017). Selective activity of oleanolic and maslinic acids on the amastigote form of *Leishmania* spp.. Iran. J. Pharm. Res..

[64] Gnoatto S.C.B., Vechia Dalla L., Lencina C.L., Dassonville-Klimpt A., Da Nascimento S., Mossalayi D., Guillon J., Gosmann G., Sonnet P. (2008). Synthesis and preliminary evaluation of new ursolic and oleanolic acids derivatives as antileishmanial agents. J. Enzyme Inhib. Med. Chem..

[65] Camacho M.D.R., Mata R., Castaneda P., Kirby G.C., Warhurst D.C., Croft S.L., Phillipson J.D. (2000). Bioactive compounds from *Celaenodendron mexicanum*. Planta Med..

[66] Tan N., Kaloga M., Radtke O.A., Kiderlen A.F., Öksüz S., Ulubelen A., Kolodziej H. (2002). Abietane diterpenoids and triterpenoic acids from *Salvia cilicica* and their antileishmanial activities. Phytochemistry.

[67] Tiwari N., Gedda M.R., Tiwari V.K., Singh S.P., Singh R.K. (2017). Limitations of current therapeutic options, possible drug targets and scope of natural products in control of leishmaniasis. Mini-Rev. Med. Chem..

[68] De Souza W., Rodrigues F.C.J. (2009). Sterol biosynthesis pathway as target for anti-trypanosomatid drugs. Interdiscip. Perspect. Infect. Dis..

[69] McCall L.-I., El-Aroussi A., Yong Choi J., Vieira D.F., De Muylder G., Johnston J.B., Chen S., Kellar D., Siqueira-Neto J.L., Roush W.R. (2015). Targeting ergosterol biosynthesis in *Leishmania donovani*: Essentiality of sterol 14α-demethylase. PLoS Negl. Trop. Dis..

[70] Souza M.T., Rocha G.C., Costa S.D., Rodrigues C.M., Ferreira C., Tavares M.T., Saraiva E., Parise-filho R., Braden H., Delorenzi J.C. (2016). Oleanolic acid (OA) as an antileishmanial agent: Biological evaluation and in silico mechanistic insights. Parasitol. Int..

[71] Warfield J., Setzer W.N., Ogungbe I.V. (2014). Interactions of antiparasitic sterols with sterol 14α-demethylase (CYP51) of human pathogens. Springer Plus.

[72] Goad L.J., Holz G.G., Beach D.H. (1984). Sterols of *Leishmania* species, implications for biosynthesis. Mol. Biochem. Parasitol..

[73] Yao C., Wilson M.E. (2016). Dynamics of sterol synthesis during development of *Leishmania* spp. parasites to their virulent form. Parasites Vectors.

[74] Begum S., Zehra S.Q., Siddiqui B.S., Fayyaz S., Ramzan M. (2008). Pentacyclic triterpenoids from the aerial parts of *Lantana camara* and their nematicidal activity. Chem. Biodivers..

[75] Qamar F., Begum S., Raza S.M., Wahab A., Siddiqui B.S., Begum S., Raza S.M. (2005). Nematicidal natural products from the aerial parts of *Lantana camara* Linn. Nat. Prod. Res..

[76] Saleh M., Kamel A., Li X., Swaray J. (1999). Antibacterial triterpenoids isolated from *Lantana camara*. Pharm. Biol..

[77] Ishibashi M., Oda H., Mitamura M., Okuyama E., Komiyama K., Kawaguchi K., Watanabe T., de Mello Alves S., Maekawa T., Ohtsuki K. (1999). Casein kinase II inhibitors isolated from two Brazilian plants *Hymenaea parvifolia* and *Wulffia baccata*. Bioorganic Med. Chem. Lett..

[78] Innocent E., Joseph C.C., Gikonyo N.K., Moshi M.J., Nkunya M.H.H., Hassanali A. (2008). Mosquito larvicidal constituents from *Lantana viburnoides* sp. *viburnoides* var *kisi* (A. rich) Verdc (Verbenaceae). J. Vector Borne Dis..

[79] Mohamed N.M., Makboul M.A., Farag S.F., Jain S., Jacob M.R., Tekwani B.L., Ross S.A. (2016). Triterpenes from the roots of *Lantana montevidensis* with antiprotozoal activity. Phytochem. Lett..

[80] National Center for Biotechnology Information Lantadene B Toxicity. https://pubchem.ncbi.nlm.nih.gov/compound/15560077#section=Non-Human-Toxicity-Excerpts.

[81] Sharma S., Sharma O.P., Singh B., Bhat T.K. (2000). Biotransformation of lantadenes, the pentacyclic triterpenoid hepatotoxins of lantana plant, in guinea pig. Toxicon.

