# Supplementary material for: Chemical Constituents with Leishmanicidal Activity from a Pink-Yellow Cultivar of Lantana camara var. aculeata (L.) Collected in Central Mexico
Source: Int J Mol Sci. 2019 Feb 18;20(4):872. doi: 10.3390/ijms20040872 (PMC6413047; doi:10.3390/ijms20040872)
Supplement: Supplementary file 1 [file ijms-20-00872-s001.pdf]

Supplementary Material

NMR chemical shifts of triterpenes

Lantanilic acid. t_R = 8.5 min (70% acetonitrile, 30% H_2O 0.1% TFA). NMR (700 MHz, $CDCl_3$). *δ* 5.57 (1H, hept, *J* = 1.2 Hz), *δ* 5.39 (1H, t, *J* = 3.6 Hz), *δ* 5.03 (1H, t, *J* = 3.2 Hz), *δ* 4.25 (1H, dd, *J* = 9.1, 3.2 Hz), *δ* 3.91 (1H, dd, *J* = 9.1, 1.5 Hz), *δ* 3.04 (1H, dd, *J* = 13.8, 4.0 Hz), *δ* 2.16 (1H, m), *δ* 2.15 (1H, m), *δ* 2.14 (3H, d, *J* = 1.1 Hz), *δ* 2.02 (1H, m), *δ* 1.87 (2H, td, *J* = 13.4, 3.5 Hz), *δ* 1.85 (3H, d, *J* = 1.1 Hz), *δ* 1.79 (1H, m), *δ* 1.75 (1H, m), *δ* 1.74 (1H, m), *δ* 1.69 (1H, m), *δ* 1.52 (2H, m), *δ* 1.36 (2H, m), *δ* 1.29 (1H, m), *δ* 1.23 (1H, m), *δ* 1.21 (1H, m), *δ* 1.20 (1H, m), *δ* 1.16 (3H, s), *δ* 1.03 (3H, s), *δ* 1.01 (3H, s), *δ* 0.96 (3H, s), *δ* 0.88 (3H, s), *δ* 0.77 (3H, s). *δ* 177.27 (COOH, C-28), *δ* 165.50 (COOR, C-1′), *δ* 157.50 (C=C, C-3′), *δ* 143.06 (C=C, C-13), *δ* 122.82 (C=C, C-12), *δ* 116.08 (C=C, C-2′), *δ* 98.94 (HO-C-OR, C-3), *δ* 75.35 (C-OR, C-22), *δ* 68.33 (CH_2, C-25), *δ* 50.94 (quaternary C, C-17), *δ* 50.46 (CH, C-5), *δ* 45.91 (CH_2, C-19), *δ* 42.22 (quaternary C, C-14), *δ* 42.12 (CH, C-9), *δ* 40.41 (quaternary C, C-4), *δ* 39.40 (CH, C-18), *δ* 38.88 (quaternary C, C-8), *δ* 37.93 (CH_2, C-21), *δ* 35.24 (quaternary C, C-10), *δ* 34.85 (CH_2, C-1), *δ* 33.88 (CH_3, C-29), *δ* 31.21 (CH_2, C-7), *δ* 30.51 (quaternary C, C-20), *δ* 29.37 (CH_2, C-2), *δ* 27.93 (CH_2, C-15), *δ* 27.58 (CH_3, C-4′), *δ* 27.37 (CH_3, C-23), *δ* 26.38 (CH_3, C-30), *δ* 25.46 (CH_3, C-27), *δ* 24.25 (CH_2, C-16), *δ* 23.90 (CH_2, C-11), *δ* 20.38 (CH_3, C-5′), *δ* 19.86 (CH_2, C-6), *δ* 18.40 (CH_3, C-24), *δ* 17.46 (CH_3, C-26). The ^{1}H, ^{13}C, COSY, HSQC, and HMBC spectra of lantanilic acid can be found in Delgado-Altamirano *et al.,* 2019 [1].

Camaric acid. t_R = 9 min (70% acetonitrile, 30% H_2O 0.1% TFA). NMR (700 MHz, $CDCl_3$). *δ* 6.01 (1H, qq, *J* = 7.3, 1.5 Hz), *δ* 5.41 (1H, br t, *J* = 3.4 Hz), *δ* 5.09 (1H, t, *J* = 3.2 Hz), *δ* 4.25 (1H, dd, *J* = 8.6, 2.7 Hz), *δ* 3.90 (1H, dd, *J* = 8.6, 1.5 Hz), *δ* 3.06 (1H, dd, *J* = 14.0, 4.1 Hz), *δ* 2.15 (2H, m), *δ* 2.01 (1H, s), *δ* 1.97 (3H, dq, *J* = 7.3, 1.5), *δ* 1.89 (2H, td, *J* = 13.5, 3.4 Hz), *δ* 1.81 (3H, quint, *J* = 1.5 Hz), *δ* 1.81 (1H, m), *δ* 1.76 (1H, m), 1.74 (1H, m), *δ* 1.71 (1H, m), *δ* 1.70 (1H, m), *δ* 1.52 (3H, m), *δ* 1.37 (2H, m), *δ* 1.29 (1H, m), *δ* 1.21 (1H, m), *δ* 1.20 (1H, m), *δ* 1.16 (3H, s), *δ* 1.03 (3H, s), *δ* 1.01 (3H, s), *δ* 0.97 (3H, s), *δ* 0.90 (3H, s), *δ* 0.77 (3H, s). *δ* 177.08 (COOH, C-28), *δ* 166.60 (COOR, C-1′), *δ* 143.03 (C=C, C-13), *δ* 157.50 (C=C, C-3′), *δ* 127.85 (C=C, C-2′), *δ* 122.95 (C=C, C-12), *δ* 98.78 (HO-C-OR, C-3), *δ* 76.05 (C-OR, C-22), *δ* 67.89 (CH_2, C-25), *δ* 50.91 (quaternary C, C-17), *δ* 50.49 (CH, C-5), *δ* 45.89 (CH_2, C-19), *δ* 42.22 (quaternary C, C-14), *δ* 42.12 (CH, C-9), *δ* 40.41 (quaternary C, C-4), *δ* 39.42 (CH, C-18), *δ* 38.47 (quaternary C, C-8), *δ* 38.00 (CH_2, C-21), *δ* 35.27 (quaternary C, C-10), *δ* 34.86 (CH_2, C-1), *δ* 33.84 (CH_3, C-29), *δ* 31.20 (CH_2, C-7), *δ* 30.21 (quaternary C, C-20), *δ* 29.52 (CH_2, C-2), *δ* 27.92 (CH_2, C-15), *δ* 27.39 (CH_3, C-23), *δ* 26.26 (CH_3, C-30), *δ* 25.47 (CH_3, C-27), *δ* 24.32 (CH_2, C-16), *δ* 23.90 (CH_2, C-11), *δ* 20.65 (CH_3, C-5′), *δ* 19.85 (CH_2, C-6), *δ* 18.39 (CH_3, C-24), *δ* 17.38 (CH_3, C-26), *δ* 15.80 (CH_3, C-4′). The ^{1}H, ^{13}C, COSY, HSQC, and HMBC spectra of camaric acid can be found in Delgado-Altamirano *et al.,* 2019 [1].

Lantadene B. t_R = 15.75 min (70% acetonitrile, 30% H_2O 0.1% TFA). NMR (700 MHz, $CDCl_3$). *δ* 5.56 (3H, t, *J* = 3.4 Hz), *δ* 5.38 (1H, t, *J* = 3.4 Hz), *δ* 5.04 (1H, t, *J* = 3.1 Hz), *δ* 3.03 (1H, dd, *J* = 13.70, 4.2 Hz), *δ* 2.55 (1H, ddd, *J* = 15.9, 11.2, 7.2 Hz), *δ* 2.37 (1H, ddd, *J* = 15.8, 6.7, 3.6 Hz), *δ* 2.13 (3H, d, *J* = 1.1 Hz), *δ* 1.92 (2H, m), *δ* 1.90 (1H, m), *δ* 1.87 (2H, m), *δ* 1.84 (3H, d, *J* = 1.1 Hz), *δ* 1.74 (1H, m), *δ* 1.71 (1H, m), *δ* 1.66 (1H, dd, *J* = 11.2, 6.4 Hz), *δ* 1.47 (1H, m), *δ* 1.41 (1H, m), *δ* 1.31 (3H, m), *δ* 1.17 (3H, s), *δ* 1.14 (1H, m), *δ* 1.09 (3H, s), *δ* 1.06 (3H, s), *δ* 1.04 (3H, s), *δ* 1.00 (3H, s), *δ* 0.88 (3H, s), *δ* 0.84 (3H, s). *δ* 217.81 (C=O, C-3), *δ* 178.72 (COOH, C-28), *δ* 165.41 (COOR, C-1′), *δ* 157.20 (C=C, C-3′), *δ* 143.22 (C=C, C-13), *δ* 122.51(C=C, C-12), *δ* 116.12 (C=C, C-2′), *δ* 75.37 (C-OR, C-22), *δ* 55.47 (CH, C-5), *δ* 50.73 (quaternary C, C-17), *δ* 47.59 (quaternary C, C-4), *δ* 47.03 (CH, C-9), *δ* 46.13 (CH_2, C-19), *δ* 42.22 (quaternary C, C-14), *δ* 39.40 (quaternary C, C-8), *δ* 39.32 (CH_2, C-1), *δ* 38.75 (CH, C-18), *δ* 37.80

(CH2, C-21), δ 36.90 (quaternary C, C-10), δ 34.24 (CH2, C-2), δ 33.88 (CH3, C-29), δ 32.43 (CH2, C-7), δ 30.21 (quaternary C, C-20), δ 27.76 (CH2, C-15), δ 27.57 (CH3, C-5′), δ 26.60 (CH3, C-23), δ 26.43 (CH3, C-30), δ 25.90 (CH3, C-27), δ 24.28 (CH2, C-16), δ 23.71 (CH2, C-11), δ 21.64 (CH3, C-24), δ 20.38 (CH3, C-4′), δ 19.69 (CH2, C-6), δ 17.00 (CH3, C-26), δ 15.30 (CH2, C-25).

Figure S1. ^{1}H NMR spectrum of Lantadene B in CDCl3.

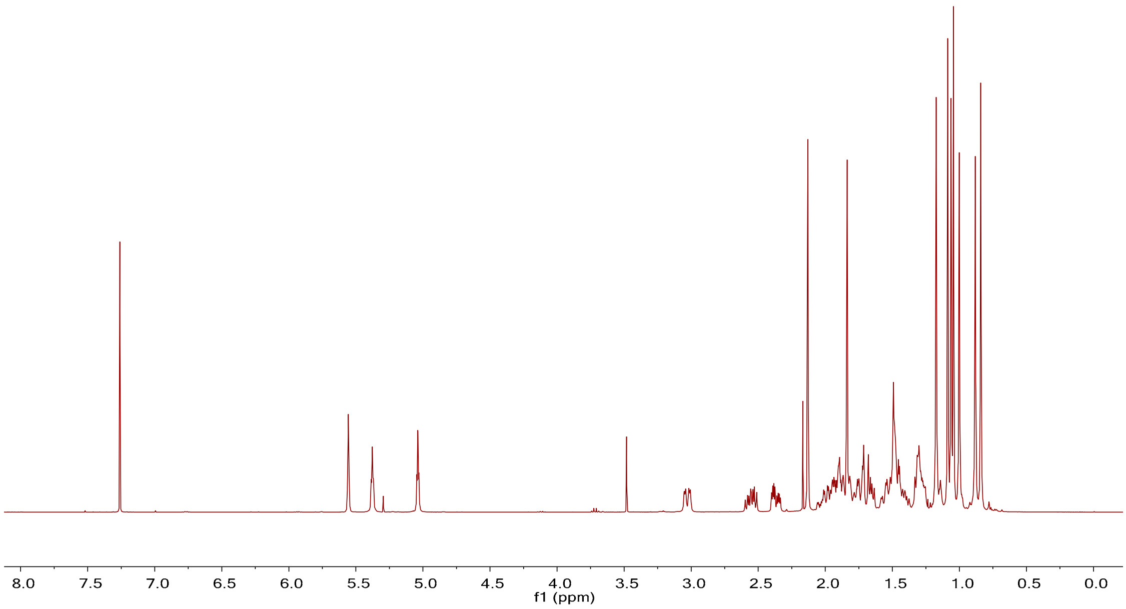

Figure S2. ^{13}C NMR spectrum of Lantadene B in CDCl3.

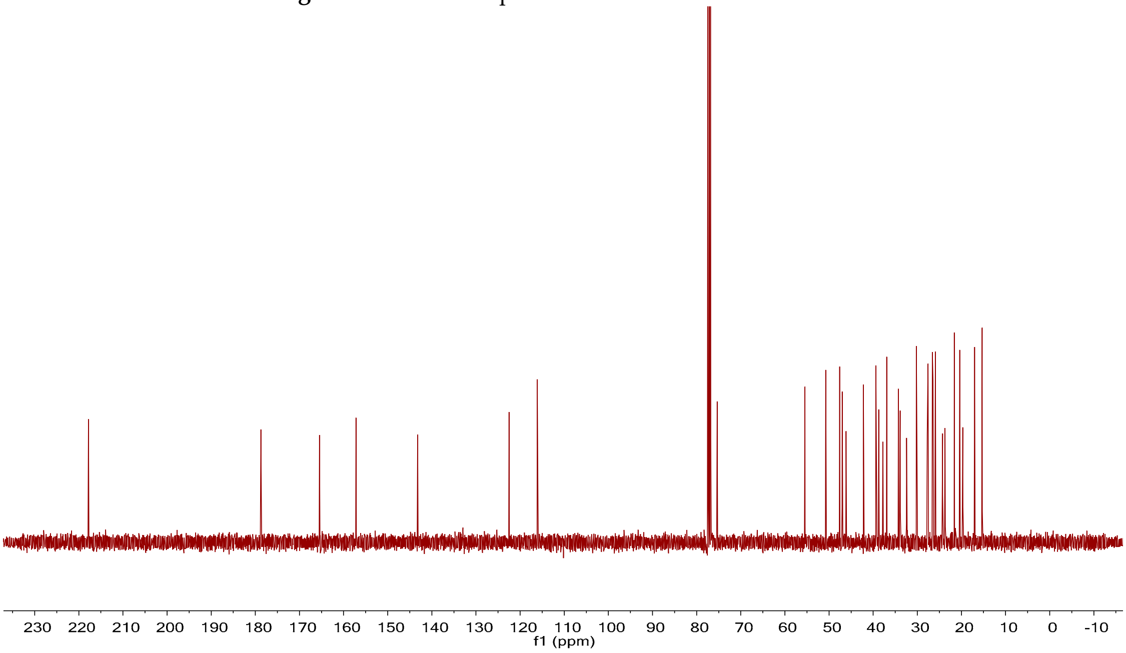

Figure S3. ^{1}H-^{1}H COSY spectrum of Lantadene B in $CDCl_3$.

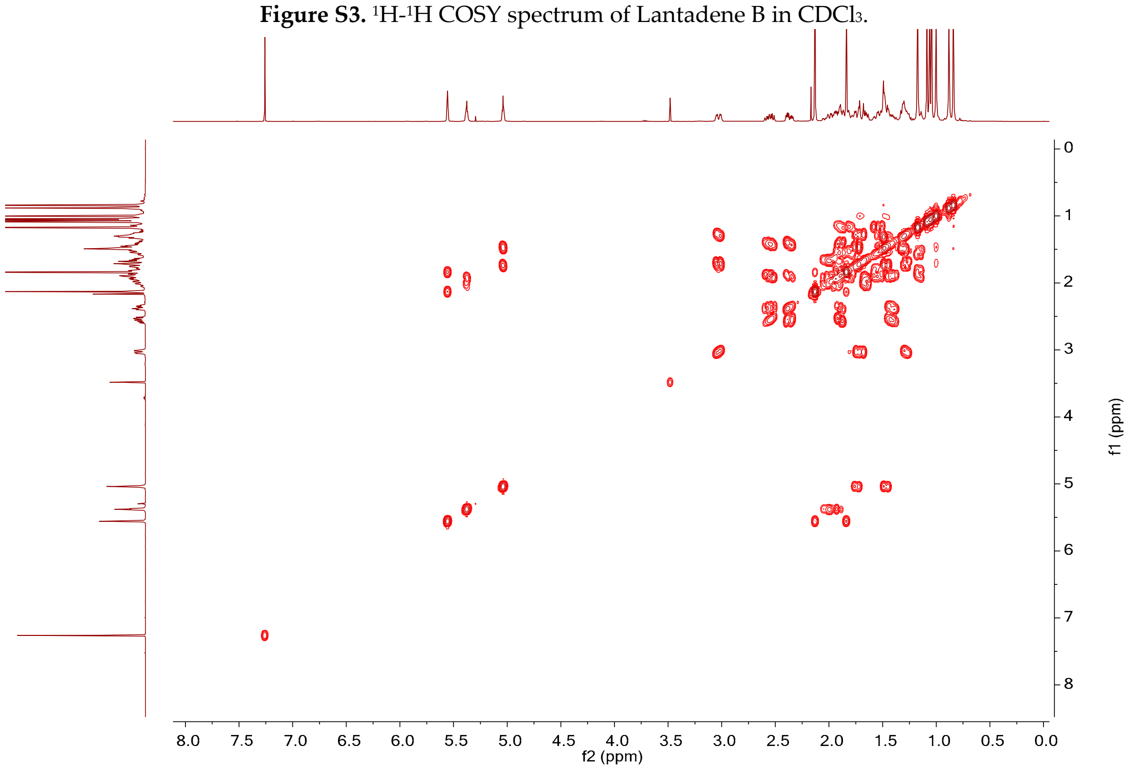

Figure S4. ^{1}H-^{13}C HSQC spectrum of Lantadene B in $CDCl_3$.

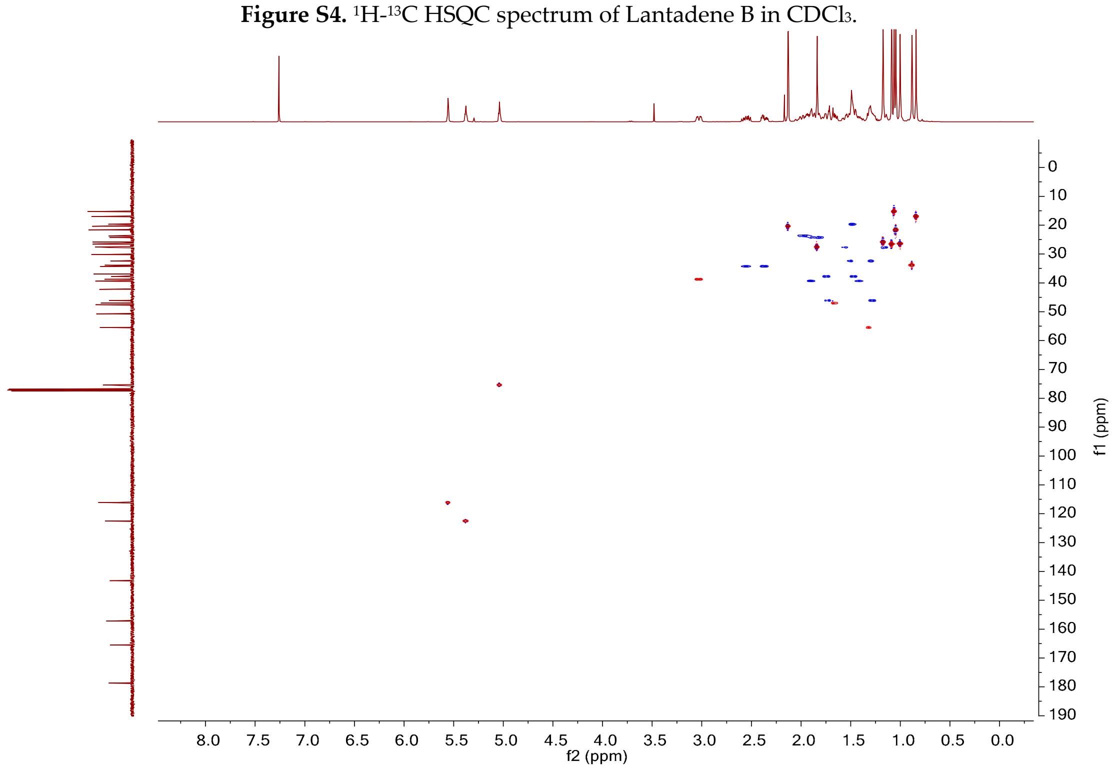

Figure S5. ^{1}H-^{13}C HMBC spectrum of Lantadene B in $CDCl_3$.

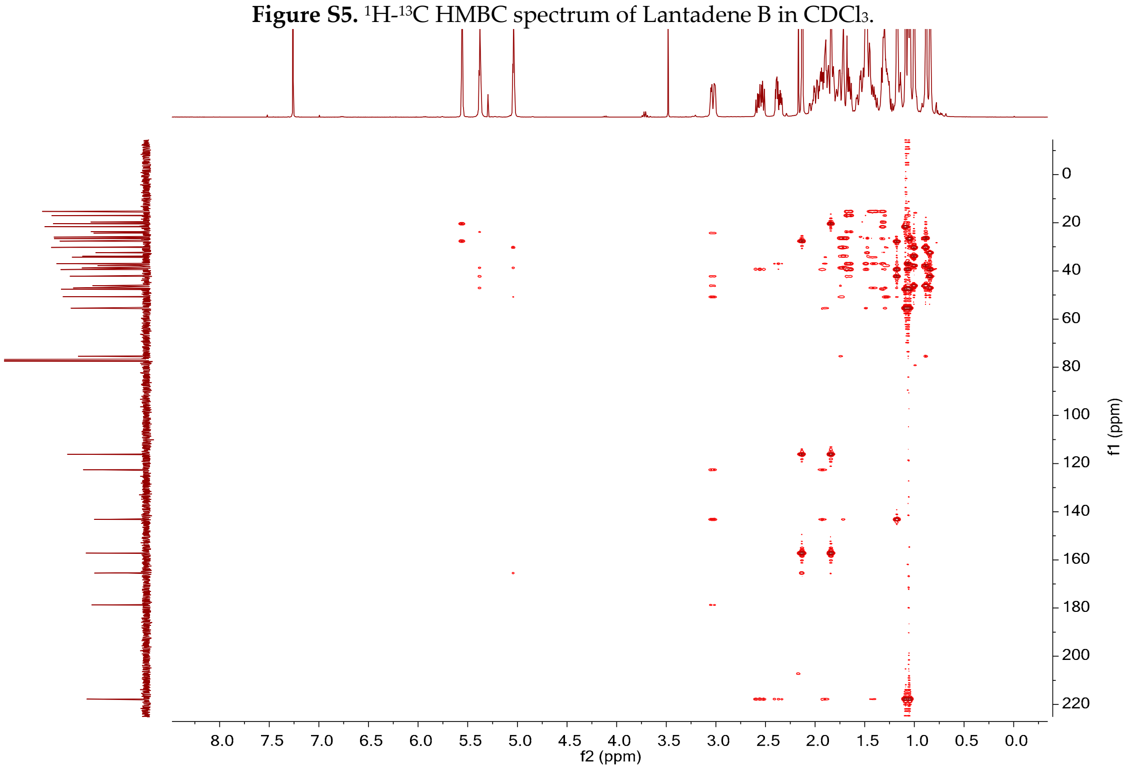

References

1. Delgado-Altamirano, R.; Rojas, A.; Esturau-Escofet, N. 1H and 13C NMR reassignment of some chemical shifts of lantanilic acid and camaric acid. *Magn. Reson. Chem.* **2019**, in Press (DOI: http://10.1002/mrc.4839).
